# *miR-223* contributes to the AGE-promoted apoptosis via down-regulating insulin-like growth factor 1 receptor in osteoblasts

**DOI:** 10.1042/BSR20150271

**Published:** 2016-04-05

**Authors:** Yi Qin, Jichao Ye, Peng Wang, Liangbin Gao, Suwei Wang, Huiyong Shen

**Affiliations:** * Department of Orthopedics, Sun Yat-Sen Memorial Hospital, Sun Yat-sen University, Guangzhou 510120, China; † Department of Orthopedics, Zhuhai People's Hospital, Zhuhai 519000, China

**Keywords:** advanced glycation end products (AGEs), apoptosis, insulin-like growth factor 1 receptor (IGF-1R), *miR-223*, osteoblasts

## Abstract

Advanced glycation end products (AGEs) have been confirmed to induce bone quality deterioration in diabetes mellitus (DM), and to associate with abnormal expression of miRNAs in DM patients or *in vitro*. Recently, miRNAs have been recognized to mediate the onset or progression of DM. In the present study, we investigated the regulation on *miR-223* level by AGE-BSA treatment in osteoblast-like MC3T3-E1 cells, with real-time quantitative PCR assay. And then we examined the inhibition of insulin-like growth factor 1 receptor (IGF-1R) expression by *miR-223*, via targeting of the 3′ UTR of IGF-1R with real-time quantitative PCR, western blotting and luciferase reporter assay. Then we explored the regulation of *miR-223* and IGF-1R levels, via the lentivirus-mediated *miR-223* inhibition and IGF-1R overexpression in the AGE-BSA-induced apoptosis in MC3T3-E1 cells. It was demonstrated that AGE-BSA treatment with more than 100 μg/ml significantly up-regulated *miR-223* level, whereas down-regulated IGF-1R level in MC3T3-E1 cells. And the up-regulated *miR-223* down-regulated IGF-1R expression in both mRNA and protein levels, via targeting the 3′ UTR of *IGF-1R*. Moreover, though the AGE-BSA treatment promoted apoptosis in MC3T3-E1 cells, the IGF-1R overexpression or the *miR-223* inhibition significantly attenuated the AGE-BSA-promoted apoptosis in MC3T3-E1 cells. In summary, our study recognized the promotion of *miR-223* level by AGE-BSA treatment in osteoblast-like MC3T3-E1 cells. The promoted *miR-223* targeted IGF-1R and mediated the AGE-BSA-induced apoptosis in MC3T3-E1 cells. It implies that *miR-223* might be an effective therapeutic target to antagonize the AGE-induced damage to osteoblasts in DM.

## Abstract (precis)

*miR-223* inhibits the advanced glycation end product (AGE)-promoted apoptosis in osteoblasts.

## INTRODUCTION

Diabetes mellitus (DM) is a global metabolic disorder, which is characterized with hyperglycaemia and hyperglycaemia-associated complications. And various molecules important for the initiation and progression of DM have been recognized. In particular, global proteome profiles of tissue and serum samples from T2DM patients or mouse models have identified lots of proteins, which are associated with the pathogenesis of T2DM [1–4]. miRNAs are a group of small and non-coding small RNA molecules that bind to the 3′ UTRs of target genes and regulate their expression predominantly at the post-transcriptional level [5,6]. Recently, miRNAs has been extensively recognized to implicate in the pathogenesis of such diseases as cancers [7] and also DM [7,8]. miRNAs are abnormally expressed in insulin-targeted organs (liver, muscle and adipose tissues) [9,10] or circulating blood [8]. And the abnormally-expressed miRNAs mediate or indicate the onset/progression of DM.

Advanced glycation end products (AGEs) develop mainly via the Maillard reaction [11] and are extremely accumulated in DM [12,13]. And AGEs have been confirmed to contribute to the pathogenesis of multiple diabetic complications such as diabetic retinopathy [14], diabetic nephropathy [15] and other micro- or marcovascular complications [16,17]. AGEs are also reported to be associated with the increased risk of bone fracture and delayed fracture healing [18,19]. In addition, AGEs promote intracellular reactive oxygen species (ROS), the mitogen-activated protein kinase (MAPK) cascade, activate nuclear factor-κB (NF-κB) and AP1 [20–22] and thus promote the apoptosis [23–25] in various types of cells [19,26]. Therefore, the AGEs pose important deregulation on the bone fracture healing in the context of DM.

Far more recently, it has been indicated that AGEs are associated with several miRNAs as *miRNA-146a* [27], *miRNA-214* [28] and *miRNA-223* (*miR-223*) [29] in DM patients or *in vitro*. *miRNA-214* was recognized to target phosphatase and tensin homologue (PTEN) and mediated the AGE-induced apoptosis in monocytes [28]. And the platelet-secreted *miR-223* regulated the endothelial cell apoptosis which was induced by AGEs via targeting the insulin-like growth factor 1 receptor (IGF-1R) [29]. However, it remains to be elucidated whether miRNAs such as *miR-223* play a role in the AGE-induced functional changes of osteoblasts.

In the present study, we investigated the *miR-223* promotion by AGE-BSA treatment in osteoblast-like MC3T3-E1 cells, and examined the targeting of the 3′ UTR of *IGF-1R* and the inhibition of IGF-1R expression by *miR-223*. Then we explored the association of *miR-223* promotion with the AGE-BSA-induced apoptosis in MC3T3-E1 cells.

## EXPERIMENTAL

### MC3T3-E1 cell culture and treatment

Mouse osteoblastic MC3T3-E1 cells (A.T.C.C.) were cultured in RPMI 1640 medium (Invitrogen), supplemented with 10% FBS (Gibco) in a humidified incubator with 5% CO_2_ at 37°C. Glycoaldehyde-AGE-BSA (AGE-BSA) was purchased from BioVision, with more than 70-folds of glycation than BSA (Sigma–Aldrich), and was dissolved in PBS (pH 7.4) with a concentration of 10 mg/ml before use. For the AGE-BSA treatment of MC3T3-E1 cells, AGE-BSA or BSA was diluted or was dissolved in RPMI 1640 medium with 2% FBS, with a final concentration of 0, 50, 100, 200 or 400 μg/ml. Then MC3T3-E1 cells which were seeded in a plate with more than 80% confluence were incubated with the AGE-BSA- or BSA-contained RPMI 1640 medium with 2% FBS for 0, 6, 12, 24 or 48 h. To promote the *miR-223* level in the MC3T3-E1 cells with or without AGE-BSA treatment, *miR-223* mimics (mature sequence: 5′-ugucaguuugucaaauacccca-3′, Sigma–Aldrich) or control miRNA (miR-Con; mature sequence: 5′-ugggcguauagacguguuacac-3′, Sigma–Aldrich) were transiently transfected with Lipofectamine RNAiMax (Invitrogen) for 12 or 24 h. To overexpress IGF-1R in MC3T3-E1 cells, the IGF-1R CDS (coding sequence) was cloned into the pcDNA3.1(+) vector (Invitrogen) with GST as negative control. And the IGF-1R-pcDNA3.1(+) or control GST-pcDNA3.1(+) plasmid was transfected into MC3T3-E1 cells by lipofectamine 2000 (Invitrogen). To abrogate the *miR-223*, MC3T3-E1 cells were infected with the lentivirus-mediated *miR-223* inhibitor or the Lenti-Con virus (both were synthesized and were packed by Sigma–Aldrich with a multiplicity of infection (MOI) of 2 or 5.

### Real-time quantitative PCR analysis of miRNA and mRNA samples

miRNA samples from MC3T3-E1 cells were prepared using the mirVana™ miRNA Isolation Kit (Ambion) under the guidance of the product's manual. And the quantitative analysis of *miR-223* level was performed with the NCode™ EXPRESS SYBR® GreenER™ miRNA qRT-PCR Kit (Thermo Fisher Scientific) according the manufacturer's guidance. Cellular mRNA was isolated with the TRIzol reagent (Life Technologies) and was supplemented with RNasin® Plus RNase Inhibitor (Promega). The quantitative analysis of IGF-1R mRNA level was performed with the Takara One Step RT-PCT kit (Takara). The quantification of *miR-223* or IGF-1R was relative presented with U6 or β-actin as reference gene, via the ∆∆Ct method [30].

### Western blot analysis of IGF-1R

3 x 10^5^ MC3T3-E1 cells post-treatment were lysed with the ice-cold Cell Lysis Buffer (Cell Signaling Technology Inc.); then the cell lysate was centrifuged with 13600 ***g*** at 4°C for 30 min and was quantified using BCA Protein Assay Reagent Kit (Pierce). Each protein sample was successively subject to the electrophoresis with 10% SDS/PAGE gradient gel, then transfer to nitrocellulose membrane (Millipore), to the blockage for non-specific binding sites on the membrane with 5% skimmed milk (4°C overnight), to the incubation (room temperature for 2 h) with the rabbit polyclonal antibody to IGF-1R (Abcam) or to β-actin (Sigma–Aldrich), and then to the incubation (room temperature for 1 h) with the incubation with horseradish peroxidase-linked secondary antibodies (Pierce). And the specific binding band to IGF-1R or to β-actin was then quantified with ECL detection systems (Thermo Scientific).

### Luciferase reporting assay

The 3′ UTR of IGF-1R containing the *miR-223*-targeted sequence (5′-aacugaca-3′) was amplified from human chromosomal DNA and was cloned into the pGL3-luciferase basic vector (Promega). The IGF-1R^mut^ Reporter containing the *miR-223*-non-targeted sequence (5′-UUGACUGU-3′) was also constructed as a control reporter plasmid. For the luciferase assay, MC3T3-E1 cells were transfected with 25 or 50 nM *miR-223* mimics or miR-Con, following the transfection with the IGF-1R reporter plasmid or the IGF-1R^mut^ Reporter plasmid via lipofectamine 2000 (Invitrogen). Twenty-four hours post-transfection, Dual luciferase re-porter assay kit (Promega) was utilized to measure luciferase activity in MC3T3-E1 cells.

### MTT assay, apoptosis assay and caspase 3 activity assay

Viability of MC3T3-E1 cells was evaluated with MTT assay. In brief, MC3T3-E1 cells seeded in 96-well plate with 85% confluence were treated with 0, 50, 100, 200 or 400 μg/ml AGE-BSA or with 200 μg/ml BSA for 24 or 48 h and were added with the MTT buffer with a final concentration of 5 mg/ml and were incubated at 37°C for 4 h. Post the removal of cell supernatant, cells then were added with 200 μl DMSO, and the *D*_570_ value was measured at 570 nm wavelength using a microplate reader (Bio-Rad Laboratories). Cellular viability was presented as a percent level to control.

The AGE-BSA-induced apoptosis of MC3T3-E1 cells was evaluated with an Annexin V-FITC Apoptosis Detection Kit (Abcam). Briefly, the AGE-BSA- or BSA-treated MC3T3-E1 cells, with or without the transfection with IGF-1R-pcDNA3.1(+) or control GST-pcDNA3.1(+) plasmid, with the infection with 2 or 5 MOI of Lenti-*miR-223* inhibitor or Lenti-Con virus were collected and were suspended in the 400 μl 1× binding buffer at a concentration of 5×10^5^ cells/ml, then 5 μl of Annexin V-FITC and propidium iodide was added in the cell suspension with a incubation at dark for 10 min; and then cells were performed flow cytometry analysis. The apoptotic cells were presented as a percentage to total cells. The caspase 3 activity was performed with the caspase 3 activity assay kit (Roche Diagnostics GmbH) according to the manual. And the activity was expressed as a relative value to control.

### Statistical analysis

Statistical analyses were performed using GraphPad Prism 6 (GraphPad Software). The *miR-223* level, IGF-1R in mRNA or protein level, the relative luciferase activity, the percentage of apoptotic cells, the caspase 3 activity between two groups were analysed by Student's *t* test. A *P* value <0.05 or less was considered significance.

## RESULTS

### Up-regulation of *miR-223* and down-regulation of IGF-1R by AGE-BSA in osteoblast-like MC3T3-E1 cells

**Figure 1:**
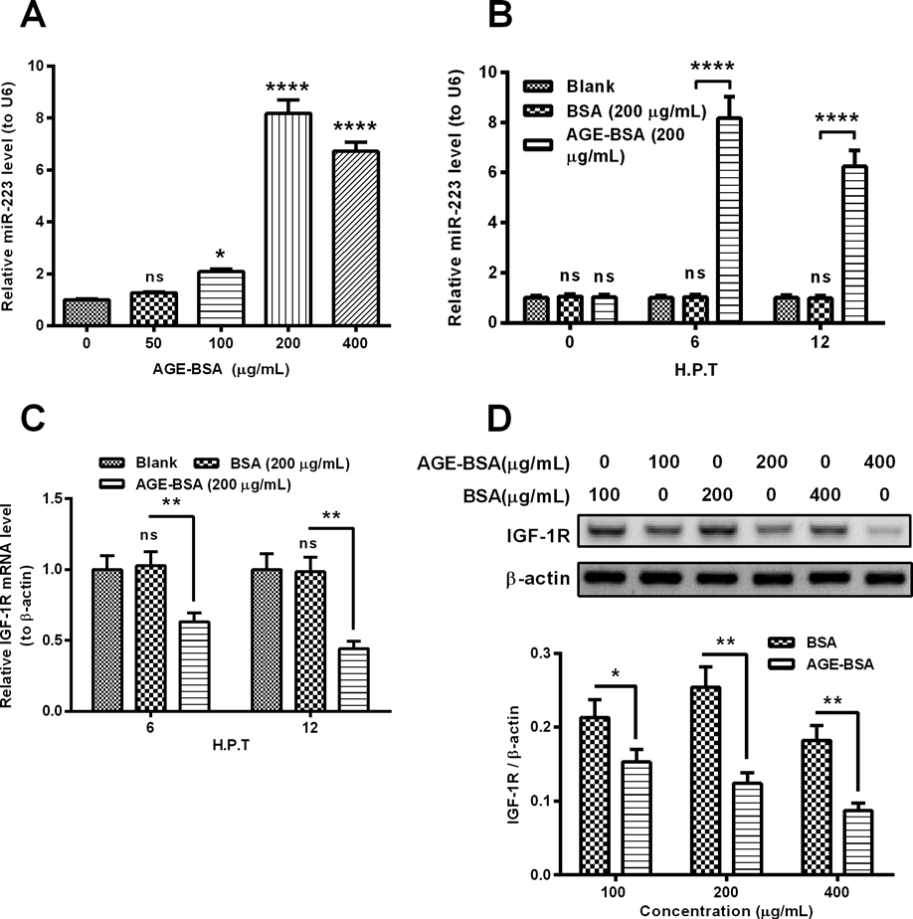
AGE-BSA treatment up-regulates *miR-223* level, whereas down-regulates IGF-1R level in osteoblast-like MC3T3-E1 cells (**A**) MC3T3-E1 cells were treated with 0, 50, 100, 200 or 400 μg/ml AGE-BSA for 6 h, then the *miR-223* level was assayed, with RT-qPCR, with U6 as a reference; (**B**) *miR-223* level in MC3T3-E1 cells which were treated with or without 200 μg/ml AGE-BSA or BSA for 6 or 12 h; (**C**) mRNA level of IGF-1R in MC3T3-E1 cells which were treated with or without 200 μg/ml AGE-BSA or BSA for 6 or 12 h; (**D**) western blot analysis of IGF-1R in protein level in the MC3T3-E1 cells post the treatment with 100, 200 or 400 μg/ml AGE-BSA or BSA for 24 h. Each data were averaged for triple independent result, ns: no significance, *P<0.05, **P<0.01 or ^****^P<0.0001.

To investigate the regulation by AGEs on the *miR-223* expression in osteoblasts, we incubated MC3T3-E1 cells with serially-diluted AGE-BSA and examined the *miR-223* level with quantitative PCR method. As shown in Figure 1A, the AGE-BSA treatment with more than 100 μg/ml significantly up-regulated the *miR-223* level in the MC3T3-E1 cells (*P*<0.01 or *P*<0.0001). And such up-regulation was confirmed in either 6 or 12 h post-treatment (H.P.T.) for 100 μg/ml AGE-BSA (either *P*<0.0001, Figure 1B). It was reported that *miR-223* targeted and inhibited IGF-1R in several types of cells [31–33]; we then examined the IGF-1R expression in both mRNA and protein levels in the AGE-BSA-treated MC3T3-E1 cells. Figure 1C demonstrated that compared with the treatment with BSA (200 μg/ml), the AGE-BSA treatment (200 μg/ml) markedly down-regulated the IGF-1R mRNA level at either 6 or 12 H.P.T. (*P*<0.01 for 0.6317±0.0643 compared with 1.0288±0.0967 at 6 H.P.T. or *P*<0.01 for 0.4436±0.0521 compared with 0.9856±0.1015 at 12 H.P.T.). And the western blotting assay confirmed the down-regulation of IGF-1R in the AGE-BSA-treated MC3T3-E1 cells. The relative protein level of IGF-1R was markedly down-regulated in the MC3T3-E1 cells treated with 100, 200 or 400 μg/ml AGE-BSA (*P*<0.05 or *P*<0.01, Figure 1D). Thus, we found the up-regulation of *miR-223* and the down-regulation of IGF-1R in AGE-BSA-treated MC3T3-E1 cells.

### *miR-223* targets and inhibits IGF-1R in MC3T3-E1 cells

**Figure 2:**
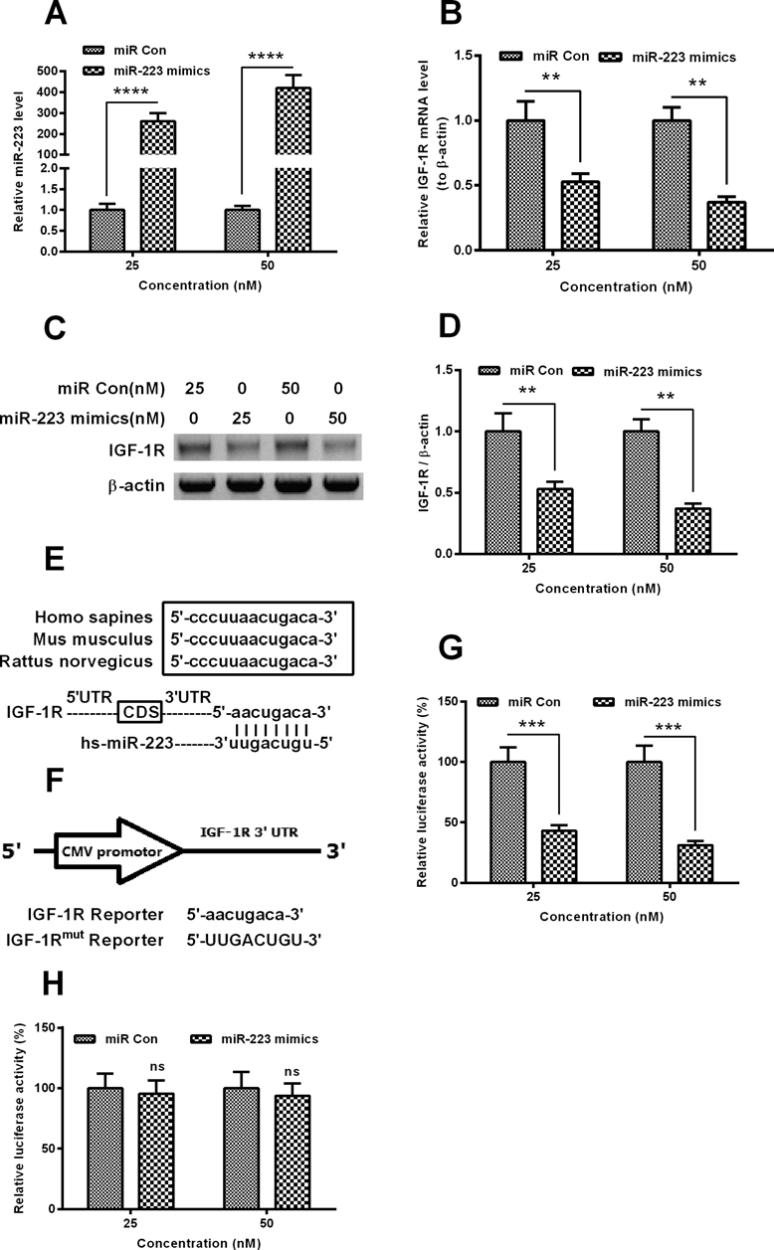
*miR-223* targets the 3′ UTR of *IGF-1R* gene and inhibits the IGF-1R expression (**A**) *miR-223* level in MC3T3-E1 cells which were transfected with 25 or 50 nM *miR-223* mimics or scramble miRNA (miR-Con) for 12 h; (**B**) mRNA level of IGF-1R in the *miR-223* mimics- or miR-Con-transfected MC3T3-E1 cells (25 or 50 nM for 12 h); (**C** and **D**) protein level of IGF-1R, by western blotting, in the *miR-223* mimics- or miR-Con-transfected MC3T3-E1 cells (25 or 50 nM for 24 h), with β-actin as control; (**E**) alignment of the consensus target sequence with IGF-1R 3′ UTR from *H. sapiens*, *M. musculus* and *R. norvegicus* with *Homo sapiens miR-223* (hs-*miR-223*); (**F**) schematic presentation of a luciferase reporter with the IGF-1R 3′ UTR. The IGF-1R 3′ UTR or the mutant IGF-1R 3′ UTR was inserted behind the cytomegalovirus promoter; (**G** and **H**) relative luciferase of the reporter with the 3′ UTR of IGF-1R (**G**) or the mutant 3′ UTR of IGF-1R (**H**) with *Renilla* luciferase as internal control, in MC3T3-E1 cells, post the *miR-223* mimics or scrambled oligonucleotide transfection. Each data were averaged for triple independent result. Statistical significance was considered when *P*<0.05 or less, ns: no significance, **P<0.01, ***P<0.001 or ^****^P<0.0001.

To associate the *miR-223* up-regulation with the down-regulated IGF-1R, and to confirm the targeted inhibition of IGF-1R by *miR-223* in MC3T3-E1 cells, we then investigated the regulation by *miR-223* up-regulation on the IGF-1R expression in MC3T3-E1 cells. It was indicated in Figure 2A that the transfection with 25 or 50 nM *miR-223* mimics markedly promoted the *miR-223* level at 12 H.P.T. (*P*<0.0001 respectively). Moreover, both mRNA (*P*<0.01 for 25 or 50 nM, Figure 2B) and protein (*P*<0.01 for 25 or 50 nM, Figures 2C and 2D) levels of IGF-1R were markedly down-regulated by the transfection with 25 or 50 nM *miR-223* mimics. And to reconfirm such targeted down-regulation of IGF-1R by *miR-223*, we constructed a luciferase reporter with the 3′ UTR. Figure 2E demonstrated the high homologous 3′ UTR sequence of *IGF-1R* from *Homo sapiens*, *Mus musculus* or *Rattus norvegicus*; and the sequence of the IGF-1R reporter, with *miR-223* target, or the IGF-1R^mut^ reporter, without the *miR-223* target was confirmed by sequencing (Figure 2F). As shown in Figure 2G, the luciferase assay with IGF-1R reporter demonstrated that the transfection with either 25 or 50 nM *miR-223* dramatically down-regulated the luciferase activity than the miR-Con (either *P*<0.001), whereas there was no such difference between *miR-223* and miR-Con, in the context of the luciferase assay with IGF-1R^mut^ reporter. Therefore, we confirmed the targeting inhibition of IGF-1R expression by *miR-223* in MC3T3-E1 cells.

### AGE-BSA treatment promotes apoptosis in MC3T3-E1 cells

**Figure 3:**
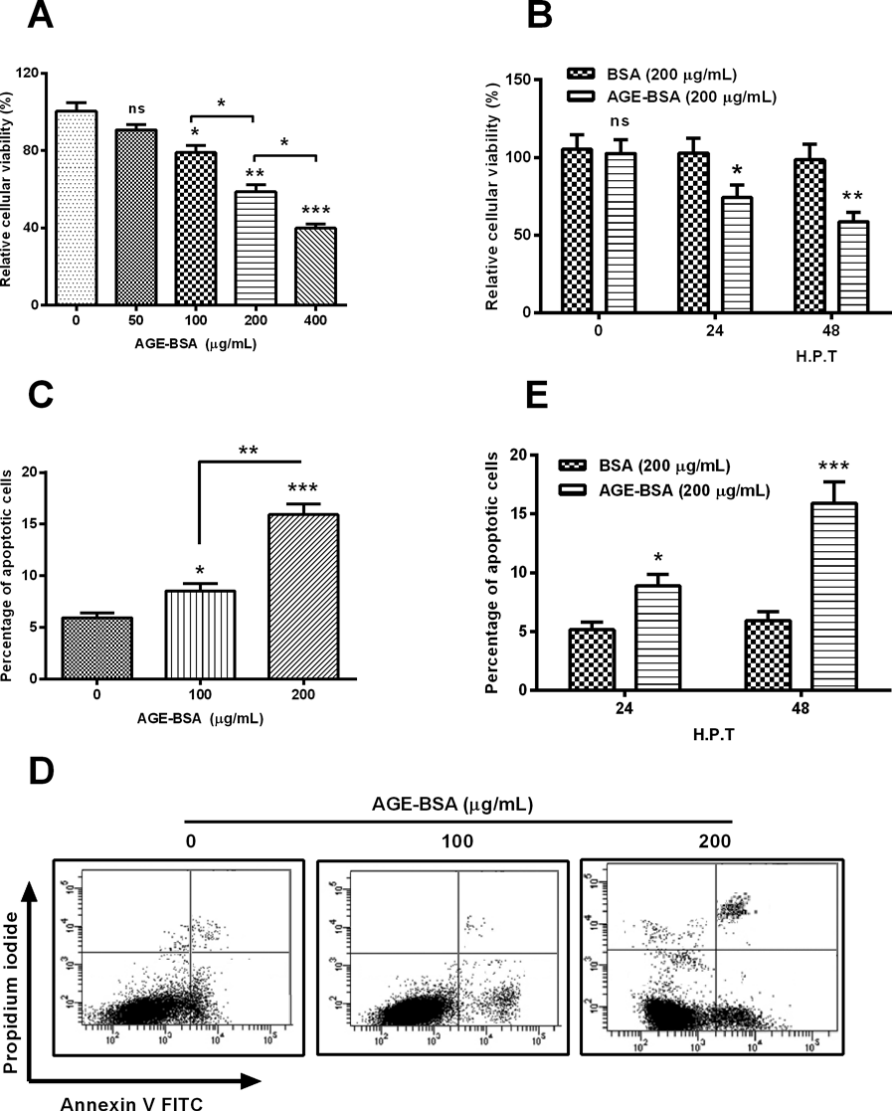
AGE-BSA reduces the viability and induces apoptosis in MC3T3-E1 cells (**A**) MC3T3-E1 cells were treated with 0, 50, 100, 200 or 400 μg/ml AGE-BSA for 48 h, then the cellular viability was examined with MTT assay; (**B**) viability of MC3T3-E1 cells which were treated with 200 μg/ml AGE-BSA or BSA for 24 or 48 h; (**C** and **D**) apoptosis of MC3T3-E1 cells which were treated with 0, 100 or 200 μg/ml AGE-BSA, revealing by the flow cytometry analysis; (**E**) apoptosis of MC3T3-E1 cells which were treated with 200 μg/ml AGE-BSA or BSA for 24 or 48 h; ns: no significance, *P<0.05, **P<0.01 or ***P<0.001.

To investigate the influence posed by AGE-BSA treatment on the cell apoptosis, MC3T3-E1 cells were subject to 50, 100, 200 or 400 μg/ml for 48 h, and the cellular viability was examined with MTT assay. Figure 3A indicated that AGE-BSA treatment with more than 100 μg/ml markedly reduced the viability of MC3T3-E1 cells (*P*<0.05, *P*<0.01 or *P*<0.001) dose-dependently (*P*<0.05). Compared with the treatment with 200 μg/ml BSA, the 200 μg/ml AGE-BSA reduced the cellular viability at either 24 or 48 H.P.T. (*P*<0.05 or *P*<0.01, Figure 3B). Moreover, we examined the apoptosis induction by AGE-BSA treatment with an Annexin V-FITC apoptosis detection kit. As indicated in Figures 3C and 3D, either 100 or 200 μg/ml AGE-BSA treatment for 48 h induced significantly high level of apoptotic cells (*P*<0.05 or *P*<0.001), also dose-dependently (*P*<0.01). And such apoptosis induction was repeatedly found at either 24 or 48 H.P.T. for 200 μg/ml AGE-BSA, rather than 200 μg/ml BSA (*P*<0.05 or *P*<0.001, Figure 3E). In summary, AGE-BSA treatment reduces cellular viability and promotes apoptosis in MC3T3-E1 cells.

### IGF-1R overexpression inhibits the AGE-BSA-promoted apoptosis in MC3T3-E1 cells

**Figure 4:**
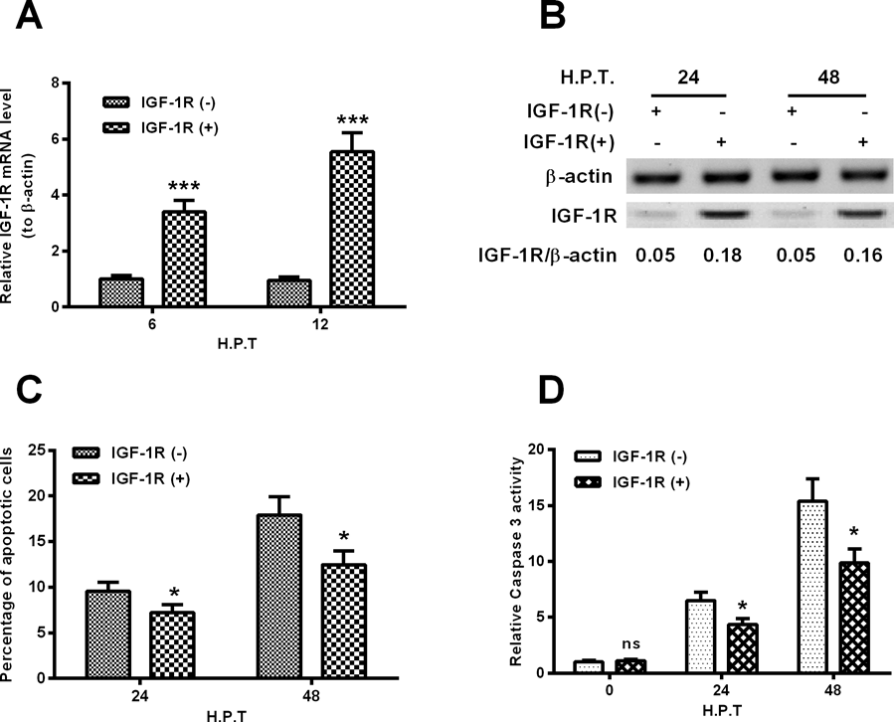
Overexpression of IGF-1R inhibits the AGE-BSA-induced apoptosis of MC3T3-E1 cells (**A**) mRNA level of IGF-1R in the MC3T3-E1 cells which were transfected with IGF-1R-pcDNA3.1(+) or GST-pcDNA3.1(+) plasmid for 6 or 12 h, in the presence of 200 μg/ml AGE-BSA; (**B**) western blot analysis of IGF-1R in protein level in the MC3T3-E1 cells which were transfected with IGF-1R-pcDNA3.1(+) or GST-pcDNA3.1(+) plasmid for 24 or 48 h, in the presence of 200 μg/ml AGE-BSA; (**C** and **D**) apoptosis (**C**) and caspase 3 activity (**D**) in the MC3T3-E1 cells which were transfected with IGF-1R-pcDNA3.1(+) or GST-pcDNA3.1(+) plasmid for 24 or 48 h, in the presence of 200 μg/ml AGE-BSA. All experiments were performed in triplicate independently and statistical significance was showed as *P<0.05 or ***P<0.001.

To explore the regulatory role of IGF-1R in the AGE-induced apoptosis of MC3T3-E1 cells, we then overexpressed IGF-1R in MC3T3-E1 cells via gain-of-function strategy. The IGF-1R coding sequence-carried IGF-1R-pcDNA3.1(+) or the control GST-pcDNA3.1(+) plasmid was transfected into MC3T3-E1 cells to manipulate the IGF-1R level. It was demonstrated that there was a significant up-regulation of IGF-1R in both mRNA (*P*<0.001 for either 6 or 12 H.P.T., Figure 4A) and protein levels (either 24 or 48 H.P.T., Figure 4B) post the transfection with IGF-1R-pcDNA3.1(+) transfection in MC3T3-E1 cells, compared with the GST-pcDNA3.1(+) transfection. In addition, the apoptotic cells and relative caspase 3 activity were also assayed in MC3T3-E1 cells which were transfected with each type of recombinant plasmid. Figure 4C demonstrated that the AGE-BSA-induced apoptosis (200 μg/ml) in MC3T3-E1 cells was markedly attenuated by the transfection with IGF-1R-pcDNA3.1(+) (*P*<0.05 for either 24 or 48 H.P.T.). And a less caspase 3 activity was induced by the 200 μg/ml AGE-BSA in the IGF-1R-up-regulated MC3T3-E1 cells (*P*<0.05 for the 24 or 48 H.P.T., Figure 4D).

### *miR-223* inhibition ameliorates the AGE-BSA-down-regulated IGF-1R and blocks the AGE-BSA-promoted apoptosis in MC3T3-E1 cells

**Figure 5:**
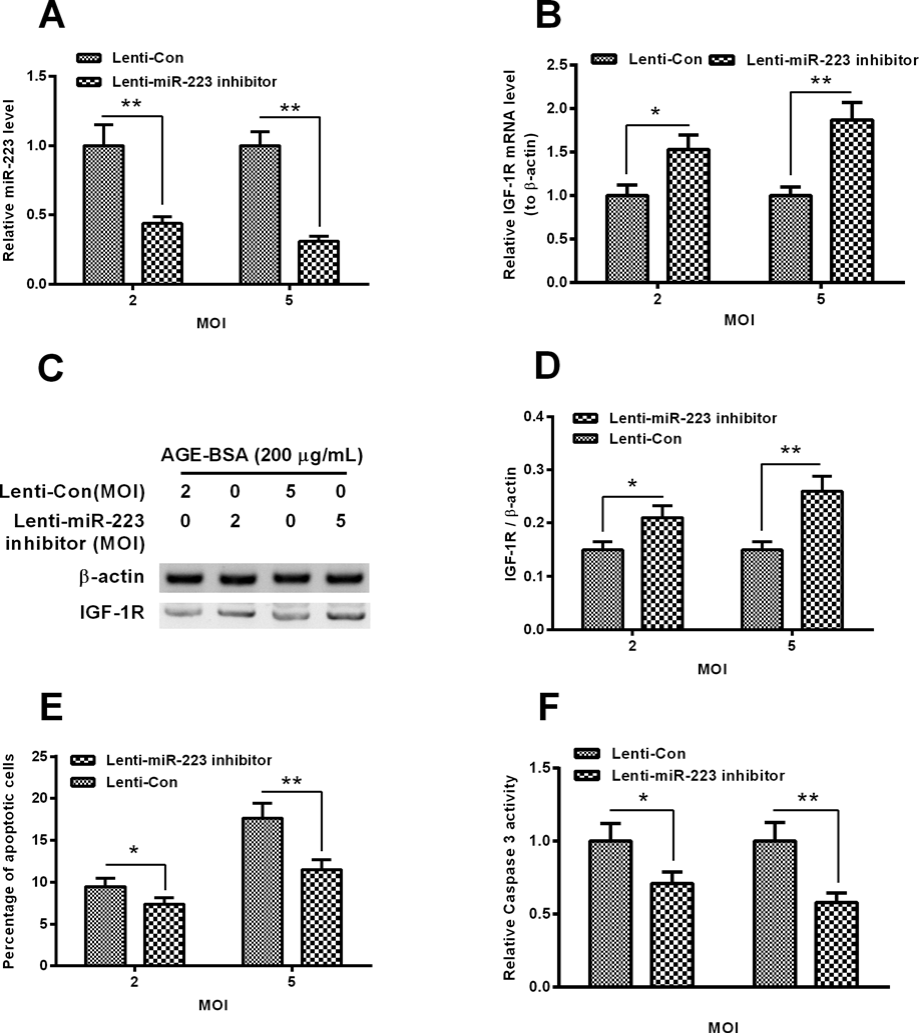
Apoptosis in the AGE-BSA-treated MC3T3-E1 cells post the *miR-223* inhibition (**A** and **B**) *miR-223* level (**A**) or the IGF-1R mRNA level (**B**) in the MC3T3-E1 cells which were infected with 2 or 5 MOI Lenti-*miR-223* inhibitor or Lenti-Con (as control) virus for 12 h, in the presence of 200 μg/ml AGE-BSA; (**C** and **D**) western blot analysis of IGF-1R in protein level (**C**) in the MC3T3-E1 cells which were infected with 2 or 5 MOI Lenti-*miR-223* inhibitor or Lenti-Con (as control) virus for 24 h, in the presence of 200 μg/ml AGE-BSA, the IGF-1R in protein level was presented as a relative level to β-actin; (**E** and **F**) apoptosis (**E**) and caspase 3 activity (**F**) in the MC3T3-E1 cells which were infected with 2 or 5 MOI Lenti-*miR-223* inhibitor or Lenti-Con virus for 48 h, in the presence of 200 μg/ml AGE-BSA. All experiments were performed in triplicate independently and statistical significance was showed as *P<0.05 or **P<0.01.

We have confirmed the targeted inhibition of IGF-1R by *miR-223* in MC3T3-E1 cells and the contribution of the reduced IGF-1R in the AGE-BSA-induced apoptosis in MC3T3-E1 cells. In order to investigate the role of the promoted *miR-223* in such apoptosis induction, we then knocked down the *miR-223* promotion with lentivirus-mediated *miR-223* inhibitor in the AGE-BSA-treated MC3T3-E1, and re-evaluated the AGE-BSA-induced apoptosis. Figure 5A confirmed the marked reduction of *miR-223* by the Lenti-*miR-223* inhibitor virus infection with 2 or 5 MOI (*P*<0.01 respectively). And the *miR-223* inhibition also blocked the IGF-1R reduction in the MC3T3-E1 cells which were treated with 200 μg/ml AGE-BSA in both mRNA (*P*<0.05 or *P*<0.01, Figure 5B) and protein (*P*<0.05 or *P*<0.01, Figures 5C and 5D) levels. Moreover, as expected, the apoptosis induction was markedly reduced by the *miR-223* inhibition with either 2 or 5 MOI Lenti-*miR-223* inhibitor virus (*P*<0.05 or *P*<0.01, Figure 5E). And the caspase 3 activity promotion by 200 μg/ml AGE-BSA was also attenuated by the Lenti-*miR-223* inhibitor virus infection with either 2 or 5 MOI (*P*<0.05 or *P*<0.01, Figure 5E). Therefore, we confirmed the mediatory role of *miR-223* in the AGE-BSA-induced apoptosis in MC3T3-E1 cells via targeting IGF-1R.

## DISCUSSION

AGEs promote apoptosis in various types of cells, such as fibroblasts, renal tubular cells and endothelial cells [34–36]. And recently, AGEs have been recognized to involve in the bone quality deterioration in DM, via promoting bone loss [37], bone fragility [38] and impairment to fracture healing [18,19]. Oxidative stress and the generation of AGEs have emerged as links between inflammation and bone destruction in DM [37]. Accumulated AGEs along with impaired collagen cross-linking and the suppression of bone turnover seem to be significant factors impairing bone strength [38]. In particular, the inflammation aroused by ROS and AGEs increases chondrocyte and osteoblast death and prolongs osteoclast survival, resulting in impaired bone regeneration in DM [19].

AGEs also up-regulated the expression of both the receptor for advanced glycation endproducts (RAGE) and galectin-3 and induced apoptosis in MC3T3-E1 cells [39]. Far more recently, AGEs have been recognized to promote several miRNAs [27–29] in DM patients or *in vitro*. In the present study, we firstly confirmed the promotion to *miR-223* by AGEs in osteoblast-like MC3T3-E1 cells. And the promoted *miR-223* contributed to the AGE-induced apoptosis in MC3T3-E1 cells. *miR-223* has been reported to associate with various types of diseases such as cancers [40,41], obesity [42], rheumatoid arthritis (RA) [43] or cardiovascular diseases [44], via targeting tumour suppressor EPB41L3 [40], targeting HSP90B1 [41] or targeting cardiac troponin I-interacting kinase [44]. Our study found the high homology of *miR-223* with the 3′ UTR of *IGF-1R* with species, such as *H. sapiens*, *M. musculus* or *R. norvegicus* for the first time. And the promoted *miR-223* by AGE-BSA treatment in MC3T3-E1 cells down-regulated the IGF-1R in both mRNA and protein levels via targeting the 3′ UTR of *IGF-1R*.

The tumour suppressive role of IGF-1R has been widely recognized in cancer cells [45,46], in stem cells [46]. And multiple regulatory roles of IGF-1R have been recognized in osteoblasts, including differentiation [47,48] and growth [49]. Our further investigation associated the AGE-induced apoptosis in MC3T3-E1 cells with the *miR-223* up-regulation and its targeted inhibition to IGF-1R. AGE-BSA treatment significantly reduced the cellular viability and induced apoptosis in MC3T3-E1 cells dose-dependently. However, the promoted apoptosis of MC3T3-E1 cells by AGE-BSA was significantly inhibited by the IGF-1R overexpression. The significantly-up-regulated IGF-1R markedly attenuated the induction by AGE-BSA of apoptosis and caspase 3 activity in MC3T3-E1 cells. Moreover, the *miR-223* inhibition also ameliorated the AGE-BSA-down-regulated IGF-1R and blocked the AGE-BSA-promoted apoptosis in MC3T3-E1 cells. The infection with lenti-*miR-223* inhibitor virus with 2 or 5 MOI blocked the IGF-1R reduction and reduced the AGE-induced apoptosis and the caspase 3 activity in MC3T3-E1 cells.

In summary, our study recognized the promotion of *miR-223* by AGE-BSA treatment in osteoblast-like MC3T3-E1 cells. The promoted *miR-223* targeted IGF-1R and mediated the AGE-BSA-induced apoptosis in MC3T3-E1 cells. Therefore, we confirmed the mediatory role of *miR-223* in the AGE-BSA-induced apoptosis in MC3T3-E1 cells via targeting IGF-1R. It implies that *miR-223* might be a effective marker to antagonize the AGE-induced damage to osteoblasts in DM.

## Abbreviations

AGE: advanced glycation end product
DM: diabetes mellitus
H.P.T.: hours post-treatment
IGF-1R: insulin-like growth factor 1 receptor
miR-Con: control miRNA
MOI: multiplicity of infection
ROS: reactive oxygen species
RAGE: the receptor for advanced glycation endproducts

## References

[1] Li R.X., Chen H.B., Tu K., Zhao S.L., Zhou H., Li S.J., Dai J., Li Q.R., Nie S., Li Y.X. (2008). Localized-statistical quantification of human serum proteome associated with type 2 diabetes. PLoS One.

[2] Rao P.V., Reddy A.P., Lu X., Dasari S., Krishnaprasad A., Biggs E., Roberts C.T., Nagalla S.R. (2009). Proteomic identification of salivary biomarkers of type-2 diabetes. J. Proteome Res..

[3] Sundsten T., Eberhardson M., Goransson M., Bergsten P. (2006). The use of proteomics in identifying differentially expressed serum proteins in humans with type 2 diabetes. Proteome Sci..

[4] Sanchez J.C., Converset V., Nolan A., Schmid G., Wang S., Heller M., Sennitt M.V., Hochstrasser D.F., Cawthorne M.A. (2002). Effect of rosiglitazone on the differential expression of diabetes-associated proteins in pancreatic islets of C57Bl/6 lep/lep mice. Mol. Cell Proteomics.

[5] Ambros V. (2004). The functions of animal microRNAs. Nature.

[6] Bartel D.P. (2004). MicroRNAs: genomics, biogenesis, mechanism, and function. Cell.

[7] Farazi T.A., Hoell J.I., Morozov P., Tuschl T. (2013). MicroRNAs in human cancer. Adv. Exp. Med. Biol..

[8] Chien H.Y., Lee T.P., Chen C.Y., Chiu Y.H., Lin Y.C., Lee L.S., Li W.C. (2015). Circulating microRNA as a diagnostic marker in populations with type 2 diabetes mellitus and diabetic complications. J. Chin. Med. Assoc..

[9] Li X., Du N., Zhang Q., Li J., Chen X., Liu X., Hu Y., Qin W., Shen N., Xu C. (2014). MicroRNA-30d regulates cardiomyocyte pyroptosis by directly targeting foxo3a in diabetic cardiomyopathy. Cell Death Dis..

[10] Carolan E., Hogan A.E., Corrigan M., Gaotswe G., O'Connell J., Foley N., O'Neill L.A., Cody D., O'Shea D. (2014). The impact of childhood obesity on inflammation, innate immune cell frequency, and metabolic microRNA expression. J. Clin. Endocrinol. Metab..

[11] Aronson D., Rayfield E.J. (2002). How hyperglycemia promotes atherosclerosis: molecular mechanisms. Cardiovasc. Diabetol..

[12] Brownlee M., Cerami A., Vlassara H. (1988). Advanced glycosylation end products in tissue and the biochemical basis of diabetic complications. N. Engl. J. Med..

[13] Brownlee M. (1995). Advanced protein glycosylation in diabetes and aging. Annu. Rev. Med..

[14] Stitt A.W., Lois N., Medina R.J., Adamson P., Curtis T.M. (2013). Advances in our understanding of diabetic retinopathy. Clin. Sci. (Lond)..

[15] Makita Z., Radoff S., Rayfield E.J., Yang Z., Skolnik E., Delaney V., Friedman E.A., Cerami A., Vlassara H. (1991). Advanced glycosylation end products in patients with diabetic nephropathy. N. Engl. J. Med..

[16] Goldin A., Beckman J.A., Schmidt A.M., Creager M.A. (2006). Advanced glycation end products: sparking the development of diabetic vascular injury. Circulation.

[17] Stirban A., Negrean M., Stratmann B., Gawlowski T., Horstmann T., Gotting C., Kleesiek K., Mueller-Roesel M., Koschinsky T., Uribarri J. (2006). Benfotiamine prevents macro- and microvascular endothelial dysfunction and oxidative stress following a meal rich in advanced glycation end products in individuals with type 2 diabetes. Diabetes Care.

[18] Dede A.D., Tournis S., Dontas I., Trovas G. (2014). Type 2 diabetes mellitus and fracture risk. Metabolism.

[19] Roszer T. (2011). Inflammation as death or life signal in diabetic fracture healing. Inflamm. Res..

[20] Cellek S. (2004). Point of NO return for nitrergic nerves in diabetes: a new insight into diabetic complications. Curr. Pharm. Des..

[21] Denis U., Lecomte M., Paget C., Ruggiero D., Wiernsperger N., Lagarde M. (2002). Advanced glycation end-products induce apoptosis of bovine retinal pericytes in culture: involvement of diacylglycerol/ceramide production and oxidative stress induction. Free Radic. Biol. Med..

[22] Ramasamy R., Vannucci S.J., Yan S.S., Herold K., Yan S.F., Schmidt A.M. (2005). Advanced glycation end products and RAGE: a common thread in aging, diabetes, neurodegeneration, and inflammation. Glycobiology.

[23] Min C., Kang E., Yu S.H., Shinn S.H., Kim Y.S. (1999). Advanced glycation end products induce apoptosis and procoagulant activity in cultured human umbilical vein endothelial cells. Diabetes Res. Clin. Pract..

[24] Liu J.P., Feng L., Zhu M.M., Wang R.S., Zhang M.H., Hu S.Y., Jia X.B., Wu J.J. (2012). The *in vitro* protective effects of curcumin and demethoxycurcumin in Curcuma longa extract on advanced glycation end products-induced mesangial cell apoptosis and oxidative stress. Planta Med..

[25] Zhou L.L., Cao W., Xie C., Tian J., Zhou Z., Zhou Q., Zhu P., Li A., Liu Y., Miyata T. (2012). The receptor of advanced glycation end products plays a central role in advanced oxidation protein products-induced podocyte apoptosis. Kidney Int..

[26] Tanaka K., Yamaguchi T., Kanazawa I., Sugimoto T. (2015). Effects of high glucose and advanced glycation end products on the expressions of sclerostin and RANKL as well as apoptosis in osteocyte-like MLO-Y4-A2 cells. Biochem. Biophys. Res. Commun..

[27] Yang Z.X., Wang Y.Z., Jia B.B., Mao G.X., Lv Y.D., Wang G.F., Yu H. (2015). Downregulation of *miR-146a*, cyclooxygenase-2 and advanced glycation end-products in simvastatin-treated older patients with hyperlipidemia. Geriatr. Gerontol. Int..

[28] Li L.M., Hou D.X., Guo Y.L., Yang J.W., Liu Y., Zhang C.Y., Zen K. (2011). Role of microRNA-214-targeting phosphatase and tensin homolog in advanced glycation end product-induced apoptosis delay in monocytes. J. Immunol..

[29] Pan Y., Liang H., Liu H., Li D., Chen X., Li L., Zhang C.Y., Zen K. (2014). Platelet-secreted microRNA-223 promotes endothelial cell apoptosis induced by advanced glycation end products via targeting the insulin-like growth factor 1 receptor. J. Immunol..

[30] Livak K.J., Schmittgen T.D. (2001). Analysis of relative gene expression data using real-time quantitative PCR and the 2(-delta delta C(T)) method. Methods.

[31] Wang Q., Zhao D.Y., Xu H., Zhou H., Yang Q.Y., Liu F., Zhou G.P. (2015). Down-regulation of microRNA-223 promotes degranulation via the PI3K/Akt pathway by targeting IGF-1R in mast cells. PLoS One.

[32] Huang K., Dong X., Sui C., Hu D., Xiong T., Liao S., Zhang H. (2014). *MiR-223* suppresses endometrial carcinoma cells proliferation by targeting IGF-1R. Am. J. Transl. Res..

[33] Jia C.Y., Li H.H., Zhu X.C., Dong Y.W., Fu D., Zhao Q.L., Wu W., Wu X.Z. (2011). *MiR-223* suppresses cell proliferation by targeting IGF-1R. PLoS One.

[34] Alikhani M., Maclellan C.M., Raptis M., Vora S., Trackman P.C., Graves D.T. (2007). Advanced glycation end products induce apoptosis in fibroblasts through activation of ROS, MAP kinases, and the FOXO1 transcription factor. Am. J. Physiol. Cell Physiol..

[35] Ishibashi Y., Matsui T., Takeuchi M., Yamagishi S. (2012). Metformin inhibits advanced glycation end products (AGEs)-induced renal tubular cell injury by suppressing reactive oxygen species generation via reducing receptor for AGEs (RAGE) expression. Horm. Metab. Res..

[36] Niiya Y., Abumiya T., Yamagishi S., Takino J., Takeuchi M. (2012). Advanced glycation end products increase permeability of brain microvascular endothelial cells through reactive oxygen species-induced vascular endothelial growth factor expression. J. Stroke Cerebrovasc. Dis..

[37] Pietschmann P., Mechtcheriakova D., Meshcheryakova A., Foger-Samwald U., Ellinger I. (2016). Immunology of osteoporosis: a mini-review. Gerontology.

[38] Poundarik A.A., Wu P.C., Evis Z., Sroga G.E., Ural A., Rubin M., Vashishth D. (2015). A direct role of collagen glycation in bone fracture. J. Mech. Behav. Biomed. Mater..

[39] Mercer N., Ahmed H., Etcheverry S.B., Vasta G.R., Cortizo A.M. (2007). Regulation of advanced glycation end product (AGE) receptors and apoptosis by AGEs in osteoblast-like cells. Mol. Cell. Biochem..

[40] Liang H., Yan X., Pan Y., Wang Y., Wang N., Li L., Liu Y., Chen X., Zhang C.Y., Gu H., Zen K. (2015). MicroRNA-223 delivered by platelet-derived microvesicles promotes lung cancer cell invasion via targeting tumor suppressor EPB41L3. Mol. Cancer.

[41] Rodriguez-Vicente A.E., Quwaider D., Benito R., Misiewicz-Krzeminska I., Hernandez-Sanchez M., de Coca A.G., Fisac R., Alonso J.M., Zato C., de Paz J.F. (2015). MicroRNA-223 is a novel negative regulator of HSP90B1 in CLL. BMC Cancer.

[42] Wen D., Qiao P., Wang L. (2015). Circulating microRNA-223 as a potential biomarker for obesity. Obes. Res. Clin. Pract..

[43] Shibuya H., Nakasa T., Adachi N., Nagata Y., Ishikawa M., Deie M., Suzuki O., Ochi M. (2013). Overexpression of microRNA-223 in rheumatoid arthritis synovium controls osteoclast differentiation. Mod. Rheumatol..

[44] Wang Y.S., Zhou J., Hong K., Cheng X.S., Li Y.G. (2015). MicroRNA-223 displays a protective role against cardiomyocyte hypertrophy by targeting cardiac troponin I-interacting kinase. Cell. Physiol. Biochem..

[45] Xu L., Qu X., Hu X., Zhu Z., Li C., Li E., Ma Y., Song N., Liu Y. (2013). Lipid raft-regulated IGF-1R activation antagonizes TRAIL-induced apoptosis in gastric cancer cells. FEBS Lett..

[46] Huang W.J., Bi L.Y., Li Z.Z., Zhang X., Ye Y. (2013). Formononetin induces the mitochondrial apoptosis pathway in prostate cancer cells via downregulation of the IGF-1/IGF-1R signaling pathway. Pharm. Biol..

[47] Joung Y.H., Lim E.J., Darvin P., Chung S.C., Jang J.W., Do P.K., Lee H.K., Kim H.S., Park T., Yang Y.M. (2012). MSM enhances GH signaling via the Jak2/STAT5b pathway in osteoblast-like cells and osteoblast differentiation through the activation of STAT5b in MSCs. PLoS One.

[48] Darvin P., Joung Y.H., Yang Y.M. (2013). JAK2-STAT5B pathway and osteoblast differentiation. JAKSTAT.

[49] DiGirolamo D.J., Mukherjee A., Fulzele K., Gan Y., Cao X., Frank S.J., Clemens T.L. (2007). Mode of growth hormone action in osteoblasts. J. Biol. Chem..

